# Association between the Intake of Different Protein Sources and Obesity Coexisting with Low Handgrip Strength in Persons near Retirement Age

**DOI:** 10.3390/nu14214684

**Published:** 2022-11-05

**Authors:** Doris Eglseer, Mariella Traxler, Silvia Bauer

**Affiliations:** Department of Nursing Science, Medical University of Graz, Universitätsplatz 4, 8010 Graz, Austria

**Keywords:** sarcopenic obesity, hand strength, obesity, retirement, protein intake, meat

## Abstract

Nutrition is important for preventing and treating sarcopenic obesity/SO, proteins play a fundamental role. This study aimed at (1) identifying the association between different protein sources, other factors, and obesity coexisting with low handgrip strength and (2) evaluating differences in protein intake between persons with coexistence of obesity with low handgrip strength, obesity alone, low handgrip strength alone and persons neither obese nor having low handgrip strength. This study is a secondary data analysis of SHARE-data among 5362 persons near retirement age. We used descriptive statistics, statistical tests and univariate and multiple logistic regression analyses. Prevalence of obesity coexisting with low handgrip strength was 4.8%. Participants with low handgrip strength had the significantly lowest intake of all protein groups, followed by participants with obesity and low handgrip strength (*p* < 0.001). Daily intake of meat/fish (0.56, CI 0.40–0.79), age (1.07, CI 1.03–1.11), two or more chronic diseases (2.22, CI 1.69–2.93), one or more limitations concerning instrumental activities of daily living (2.23, CI 1.60–3.11), and moderate activity more than once a week (0.44, CI 0.33–0.57) were significantly related factors regarding obesity coexisting with low handgrip strength. Findings suggest that a daily intake of meat/fish is associated with lower odds of suffering from obesity with low handgrip strength in retirement-aged persons. Further studies are needed for specific recommendations regarding different protein sources for obese persons with low muscle mass and/or strength.

## 1. Introduction

The process of aging is associated with changes in body composition, which includes an increase in fat mass and a simultaneous decline in muscle mass [1,2,3]. Fat mass increases until the seventh decade of life and declines thereafter. The decline in muscle mass starts earlier in life, whereas the peak of the loss of muscle mass starts around the age of 50 [4]. In addition, muscle strength also decreases with age. A decline in handgrip strength, for example, also starts as early as the fifth decade of life [5].

This means that the changes in body fat as well as muscle mass and strength occur mainly between the ages of 50 to 70. This includes the retirement phase, since the average retirement age in OECD countries varies between 48 and 67 years, although large differences are observed between some countries [4,6,7]. The transition time to retirement, therefore, is a critical phase for preventing sarcopenia, obesity and the combination of both syndromes, referred to as sarcopenic obesity (SO) [8]. 

Both sarcopenia and obesity lead to negative health consequences which may be exacerbated when both syndromes are present. One study has shown that SO predicts disability more accurately than the presence of only one of the two conditions [9]. Moreover, SO, as well as obesity coexisting with low handgrip strength, increases immobility, disability, fall rates and is related to a lower quality of life [10,11,12,13]. Furthermore, it has been associated with diseases such as osteoarthritis, dyslipidaemia, insulin resistance and cardiovascular disease [1,9,11,14]. Sarcopenic obesity affects up to 33% of older persons worldwide [1,15].

Nutrition is an important modifiable risk factor for SO [1,16]. Hence, especially the intake of proteins plays a crucial role [1], because protein enhances the anabolic activity in skeletal muscle mass and provides the necessary amino acids to stimulate muscle protein synthesis [17]. An insufficient protein intake leads to a negative nitrogen balance and results in skeletal muscle atrophy, impaired muscle growth and poor physical function in older adults [16,18,19]. Therefore, it is important to consume an adequate amount of protein to prevent wasting and to maintain skeletal muscle mass, strength and function [19]. 

There are numerous available types of protein sources which differ in their amino acid composition and absorption kinetics and therefore with regard to protein quality and anabolic effect [20,21]. Dairy products, eggs and meat include all nine essential amino acids, which is why they are classified as complete protein sources [22]. They additionally have a high leucine content which is known to increase muscle protein synthesis [23,24]. Legumes, for example, are a plant-based protein source and contain a high proportion (20–40%) of proteins, but their amino acid profile and digestibility implies that their protein quality is not as high as in animal-based protein-rich foods [21,22]. 

Overall, studies have shown that protein intake and SO are interrelated [25]. However, we still do not know how the intake of different protein sources, such as meat, dairy products, or legumes, is associated with a coexistence of obesity and low handgrip strength in persons near retirement age. This information is of relevance, since we know that older persons tend to consume less protein than is recommended [26]. Understanding more about the most effective protein sources can help healthcare providers and nutritionists to tailor recommendations for protein intake in this specific group of persons near retirement age. 

The aims of this study were (1) to identify the association between the intake of different protein sources as well as other factors and the presence of obesity coexisting with low handgrip strength and (2) to evaluate differences in the intake of different protein sources between persons with a coexistence of obesity and low handgrip strength, obesity alone and low handgrip strength alone and persons who were neither obese nor had low handgrip strength. 

## 2. Materials and Methods

### 2.1. Design

We conducted a secondary data analysis of cross-sectional data obtained from the population-based Survey of Health, Ageing and Retirement in Europe (SHARE), which is approved by the Ethics Council of the Max Plank Society in Germany [27]. The survey is performed in accordance with the ethical standards laid down in the 1964 Declaration of Helsinki and its later amendments. Written informed consent is obtained. SHARE is a research infrastructure that collects information on the health, employment and social conditions of Europeans aged 50 years and older from 27 European countries and Israel. SHARE includes non-hospitalized people who speak the native language of the respective country [28]. For the present study, data from the eighth wave were used. Data collection started in October 2019, but was interrupted due to the outbreak of the COVID-19 pandemic in March 2020. Therefore, the data collection was suspended and a new telephone-administered survey was conducted from June to August 2020. For the current analysis, we excluded 3624 persons from the initial sample of 95255 persons because they were not in the defined age range between 50 to 70 years. Furthermore, we excluded persons living in a nursing home (*n* = 25) and individuals for whom no information about their BMI (*n* = 85,912), handgrip strength (*n* = 327), or protein intake (*n* = 5) was available. The final sample consisted of 5362 participants.

### 2.2. Data Collection and Variables

For the purpose of data collection, SHARE generally uses computer-assisted personal interviews, as well as paper and pencil for drop-offs [28]. We used the information about the demographic characteristics, protein intake, BMI, hand grip strength, instrumental activities of daily living (iADL), the presence of chronic diseases and physical activity. We chose iADLs because they are important factors required to live independently, which becomes even more highly desirable as the individual ages. The iADLs consists of eight domains [29]. Participants are asked whether they have any problems with one or more of the following: “doing work around the house or garden”, “leaving the house independently/accessing transportation”, “shopping for groceries”, “doing personal laundry”, “managing money”, “preparing a hot meal”, “taking medications”, or “making telephone calls”.

Protein intake was assessed by asking the participants how often they consume different protein-rich foods. Protein sources were clustered into three categories: dairy products, meat and fish, and eggs and legumes. The five answer categories were: “every day”, “3–6 times a week”, “2 times a week”, “1 time a week” and “less than once a week”. In order to make a concrete statement about high or low intake, we decided that a daily consumption would be considered as high intake and a weekly consumption for the purposes of this analysis, regardless of whether consuming the food three to six times per week or once per week would be considered a medium/low intake.

The BMI was calculated based on the self-reported weight and height, and a high BMI was set as equal or higher than 30 kg/m^2^. A low hand gripstrength was defined as a handgrip strength below the 20th percentile of the total sample. This is proposed as an appropriate method for defining low handgrip strength by expert groups [30,31]. For our sample, this was <35 kg for men and <22 kg for women. Handgrip strength was assessed by using a handheld dynamometer (Smedley, S Dynamometer, TTM, Tokyo, 100 kg) and by following the standardized procedures. Participants were standing (preferably) or sitting with their elbow flexed at a 90° angle and the upper arm held vertically against the trunk. Participants were instructed to press with maximum effort, twice each time on the right- and left-hand side. The best try with the highest value was used for the analysis.

### 2.3. Statistical Analysis

To analyse the data, we used the IBM SPSS Statistics for Windows software package, version 27 (IBM, Corp., Armonk, NY, USA). All variables of interest were analysed descriptively. Dichotomous data are presented as absolute and relative frequencies, and continuous data are presented as means and SD or as medians and range.

To evaluate the association between the intake of different protein sources and the presence of obesity coexisting with low handgrip strength, we took a three-step approach. First, we performed chi-squared tests or Mann-Whitney U tests to identify potentially significantly associated factors for sarcopenic obesity. We chose the independent variables based on an examination of recent literature, the availability of variables in the dataset as well as on the reviewers’ clinical and research experience. Subsequently, we conducted univariate logistic regression analysis with the following variables: age, gender, 1+ IADL limitations, 2+ chronic diseases, daily intake of dairy products, daily intake of meat/fish, daily intake of eggs/legumes, moderate activity more than once a week and being retired. Prior to carrying out the third step, a multivariate logistic regression, we conducted tests to assess multicollinearity for all influencing variables based on a variance inflating factor (VIF); all of these were less than four [32]. All items for which a significant association with obesity coexisting with low handgrip strength was identified in the univariate analyses were included in the multivariate logistic regression analysis. As the intake of different protein sources was our primary topic of interest, the daily intake of all protein sources was included in the multivariate analysis regardless of whether or not it had been determined to be significant in the univariate analyses. The Hosmer-Lemeshow test, a goodness of fit test, was used to describe the fit of the model, and we calculated the odds ratio with confidence intervals of 95%. *p*-values lower than 0.05 were considered to be statistically significant.

## 3. Results

### 3.1. Patient Characteristics

In this study, 5362 participants from 27 European countries were included. Participants from Poland formed the majority of the sample (14.6%), followed by Lithuanians (14.4%), Romanians (14.3%) and Slovakians (12.5%). The mean age of the participants was 62 years, and 57% were female. Nearly half of the individuals were retired (44.1%). Another 4.5% (*n* = 238) were unemployed, and 4.4% of the participants were unable to work due to health issues. Furthermore, 2237 individuals (41.7%) suffered from two or more chronic diseases. According to the WHO BMI classification, 30.0% (*n* = 1611) were obese, 41.9% (*n* = 2246) were overweight, and 27.7% (*n* = 1484) had a normal BMI. Of these 5362 individuals, 17.5% (*n* = 936) had a low hand grip strength. The prevalence of obesity coexisting with low handgrip strength was 4.8% (*n* = 255). More detailed information about the participants’ characteristics is shown in Table 1.

### 3.2. Daily Intake of Different Protein Sources

**Figure 1:**
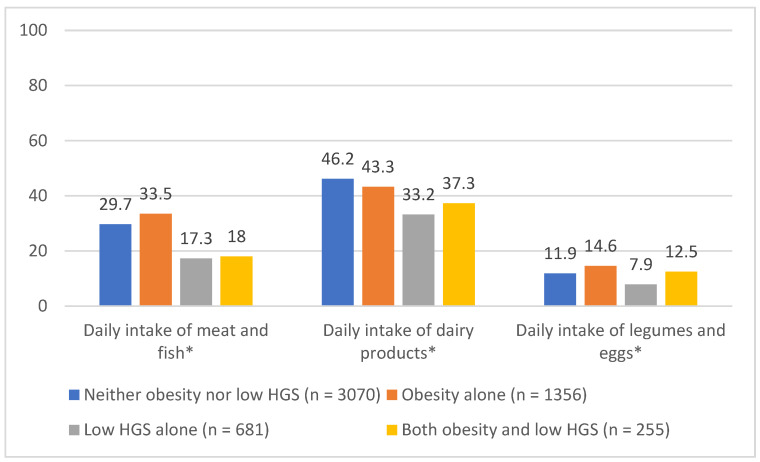
Daily intake of different protein sources according to the presence/absence of obesity coexisting with low handgrip strength, obesity alone and low handgrip strength alone; *p*-value calculated with chi-squared test; * significant difference between the groups observed.

The frequency with which different protein sources were consumed differed significantly between persons with both obesity and low handgrip strength, obesity alone, low handgrip strength alone and persons who were neither obese nor sarcopenic (*p* < 0.001). Participants with low handgrip strength alone had the significantly lowest intake of all protein-rich food groups, followed by participants with both obesity and low handgrip strength (*p* < 0.001). Figure 1 shows the detailed results.

### 3.3. Association between Protein Intake and Other Factors and Obesity Coexisting with Low Handgrip Strength

The results of the univariate logistic regression analysis revealed that older age, being retired, having two or more chronic diseases, having one or more limitations in performing instrumental activities of daily living, failing to perform moderate activity more than once a week, and failing to eat meat/fish on a daily basis are factors that are significantly associated with obesity coexisting with low handgrip strength. In the final multivariate model, the factors of age, having two or more chronic diseases and having one or more limitations in performing instrumental activities of daily living were significantly associated with higher odds of obesity with low handgrip strength, while moderate activity more than once a week and having a daily intake of meat or fish were associated with lower odds of obesity with low handgrip strength. The detailed results are shown in Table 2.

## 4. Discussion

The primary aim of this study was to investigate the association between the daily intake of different protein sources as well as other factors and the presence of obesity coexisting with low handgrip strength in persons near retirement age. Furthermore, we evaluated the differences in the intake of different protein sources between persons with/without obesity with low handgrip strength, obesity alone and low handgrip strength alone in this target group.

We found that the daily consumption of meat/fish and moderate physical activity more than once a week were associated with lower odds of obesity coexisting with low handgrip strength. Indicators that increased the risk of obesity with low handgrip strength were higher age, suffering from two or more chronic diseases and having one or more limitations in performing instrumental activities of daily living. Participants with low handgrip strength had the lowest intake of all protein groups, followed by participants with both obesity and low handgrip strength.

Our results show that the daily intake of meat/fish is associated with decreased odds of obesity coexisting with low handgrip strength. This finding is in line with those of some smaller cross-sectional studies which showed that persons following an omnivorous diet have more muscle mass and can more effectively preserve muscle mass as compared to vegetarians with the same protein intake [33,34]. Intervention studies show ambivalent results. Campbell et al. found that older men undergoing resistance training and following a meat-containing diet had more significantly increased muscle mass than older men following a vegetarian diet [35]. Daly et al. conducted a similar RCT with 100 women aged 60 to 90 and also found that the consumption of red meat increases muscle mass and strength gain during resistance training [36]. This finding could not be confirmed in another comparable study, which found that muscle mass and strength increased during a period of resistance training, regardless of whether the diet contained beef or was exclusively vegetarian [37].

The association between meat/fish consumption and higher handgrip strength in obese persons near retirement age in our study may be explained by the fact that meat contains not only all nine essential amino acids and high amounts of leucine, but also creatine which also has a potential positive effect on muscle mass and function [38]. However, meat (specifically processed meat) has also been shown to have negative influences on health and to promote certain diseases such as coronary heart diseases or cancer; its consumption has also been associated with a higher risk of mortality [39]. Professional organizations like the World Cancer Research Fund (WCRF) provide a clear recommendation to limit the consumption of processed and red meats, as these have shown to increase the risk of cancer, and especially colorectal and breast cancer [40]. The negative health effects of meat consumption do not appear to apply to white and unprocessed meat [39]. In our analysis, it was not possible to differentiate between the different types of meat (e.g., processed meat, white, or red meat). Therefore, we cannot make any statements about the intake of different types of meat and its association with the presence of obesity coexisting with low hand grip strength.

Experts propose that meat should be included in the diet 4–5 times a week, preferring white meat to red meat and trying to avoid processed meat, in order to help prevent sarcopenia [41]. This may also be appropriate for persons with coexisting obesity with low hand grip strength or persons with sarcopenic obesity, but further studies are needed to gather evidence that provides support for the association between the different types of meat and fish as well as the amount of meat and the consequences for persons with (a risk of) sarcopenic obesity. Taking into account the already known negative impacts of processed and red meat, the recommendations for higher meat consumption should be connected to a higher intake of white meat but a moderate or lower intake of red and processed meat.

We also found that the intake of dairy products was associated with lower odds of obesity coexisting with low handgrip strength, but the relationship was significant only in the univariate regression analysis. An association between an increased dairy protein intake and sarcopenic obesity was also detected in another cohort [42]. The intake of dairy products should be given extra attention. First, this is because the amino acid leucine highly stimulates muscle protein synthesis in older adults [23,43]. However, it is unclear whether the muscle-stimulating effect of leucine can be only achieved by taking dietary supplements or also by following a normal diet without consuming supplements. Second, evidence suggests that consuming yogurt and other sour milk products improves the microbiota and reduces chronic inflammation in obese persons; this means that consuming these products may offer another health advantage for persons with sarcopenic obesity and may support weight loss [44].

In our study, we did not find an association between the intake of eggs/legumes and obesity coexisting with low handgrip strength. For legumes, this might be due to the lower protein quality of plant-based protein-rich foods [21,22]. Studies suggested that to provide an adult with the optimal amount and variety of essential amino acids and to stimulate muscle protein synthesis, they have to eat about 48 g of pea protein (for whey protein, about 32 g are sufficient) [45]. A larger amount of legumes is probably more difficult to ingest and, consequently, the average portion sizes may be insufficient to stimulate muscle protein synthesis and, in turn, improve muscle mass and strength. Compared to whey or soy proteins, proteins from legumes have not been studied extensively and there are many remaining open questions. It has to be stated that the intake of eggs and legumes was generally lower than the intake of meat, fish and dairy products in our sample.

Our study findings provide important insights into the intake of different sources of protein by a large European sample and the association between this intake and obesity coexisting with low handgrip strength. However, the study also had some limitations. First, the participants’ weights and heights were queried and not measured; this may have resulted in over- or underestimations. Second, this is a cross-sectional study and causal relationship cannot be inferred. Furthermore, this study included a European population and the results cannot be generalized to other populations, such as Asians. Third, we considered a combination of high BMI and low hand grip strength as surrogate parameters for sarcopenic obesity. BMI and handgrip strength alone do not provide information about body composition, which is in fact an important factor for diagnosing sarcopenia or sarcopenic obesity. The recently published ESPEN and EASO consensus statement [46] on sarcopenic obesity proposes taking a two-step approach to confirm sarcopenic obesity: In the first step, the muscle strength (hand grip strength) is evaluated and, in the second step, the body composition is measured. However, data on body composition were not available from the SHARE survey. This limits the comparability with other studies. Fourth, the different protein sources were grouped within the SHARE questionnaire. Therefore, no conclusions can be drawn regarding the effects of consuming single protein sources, such the types of meat, legumes, fish, or eggs. We also do not know the participants’ total protein intake, which might present a confounding factor. Furthermore, we do not know which type of exercise the participants carried out (it was asked about moderate activity in general). This information would have been useful in order to differentiate between the various exercise types.

## 5. Conclusions

The results of our analyses suggest that a higher consumption of meat/fish is associated with lower odds of a coexistence of obesity and low handgrip strength in retirement-aged persons. Other protein sources such as legumes or eggs seem to be less associated with obesity coexisting with low handgrip strength. These results underline the fact that protein intake is an important player in the complex phenomenon of sarcopenic obesity. Making any recommendations for our target group is a rather complex task, since the overall aim is not only to preserve muscle mass and strength but also to reduce obesity-related health problems. More studies are needed to examine the effects of different protein sources, not only of plant-based vs. animal-based protein sources, but also to be able to make distinctions between these two protein source groups. In the future, researchers should also focus on meat alternatives that have similar impacts on muscle metabolism but do not have the negative side effects of high meat intake.

## Figures and Tables

**Table 1:** Participants’ characteristics, shown separately for persons with and without obesity coexisting with low handgrip strength.

	All Participants (*n* = 5362)	Participants with Obesity Coexisting with Low Handgrip Strength (*n* = 255)	Participants without Obesity Coexisting with Low Handgrip Strength (*n* = 5107)	*p*-Value *
Female, *n* (%)	3054 (57.0)	153 (60.0)	2901 (56.8)	0.315
Age (y), median (range)	62 (50–70)	65 (52–70)	62 (50–70)	<0.001
Retired, *n* (%)	2.363 (44.1)	148 (58.0)	2215 (43.4)	<0.001
BMI, median (range)	27.5 (16–57)	33.5 (30–57)	27.3 (16–54)	<0.001
Total number of chronic diseases, median (range)	1 (0–10)	2 (0–10)	1 (0–10)	<0.001
Most common diseases, *n* (%)				
Hypertension	2114 (39.4)	150 (58.8)	1964 (38.5)	<0.001
Hypercholesterolemia	1085 (20.2)	64 (25.1)	1021 (20.0)	0.048
Osteoarthritis/other rheumatism	773 (14.4)	60 (23.5)	713 (14.0)	<0.001
Diabetes/high blood sugar	591 (11.0)	70 (27.5)	521 (10.2)	<0.001
Rheumatoid arthritis	351 (6.5)	24 (9.4)	327 (6.4)	0.058
Chronic lung disease	215 (4.0)	17 (6.7)	198 (3.9)	0.027
Chronic kidney disease	116 (2.2)	10 (3.9)	106 (2.1)	0.048
More than one limitation in instrumental activities of daily living, *n* (%)	422 (7.9)	58 (22.7)	364 (7.1)	<0.001
Handgrip strength, mean kg (SD)	34.1 (11.2)	21.8 (6.5)	34.7 (11.0)	<0.001
Moderate activity > 1×/week, *n* (%)	3448 (64.3)	106 (41.6)	3342 (65.4)	<0.001
Daily intake of protein sources, *n* (%)				
Dairy products	2325 (43.4)	95 (37.3)	2230 (43.7)	0.044
Meat and fish	1529 (28.5)	46 (18.0)	1483 (29.0)	<0.001
Eggs and legumes	649 (12.1)	32 (12.5)	617 (12.1)	0.823

* *p*-value between patients with and without sarcopenic obesity, calculated with chi-squared test, Mann-Whitney *U* test, or *t*-test, depending on the data.

**Table 2:** Predictors for obesity coexisting with low handgrip strength, analysed with univariate and multivariate binary logistic regression analysis.

	Univariate Logistic Regression		Multivariate Logistic Regression	
Variables	OR (95% CI)	*p*-Value	OR (95% CI)	*p*-Value
Age	1.10 (1.07–1.1)	0.000	1.07 (1.03–1.11)	0.000
Female	1.14 (0.88–1.47)	0.315		
Being retired	1.80 (1.40–2.33)	0.000	0.98 (0.70–1.36)	0.878
Having 2+ chronic diseases	2.64 (2.03–3.43)	0.000	2.22 (1.69–2.93)	0.000
Having 1+ IADL limitations	3.83 (2.81–5.23)	0.000	2.23 (1.60–3.11)	0.000
Moderate activity > 1×/wk	0.38 (0.29–0.49)	0.000	0.44 (0.33–0.57)	0.000
Daily intake of meat or fish	0.54 (0.39–0.74)	0.000	0.56 (0.40–0.79)	0.001
Daily intake of dairy products	0.77 (0.59–0.99)	0.044	0.79 (0.60–1.03)	0.085
Daily intake of eggs or legumes	1.04 (0.71–1.53)	0.823	1.50 (1.00–2.25)	0.052

OR = Odds Ratio, CI = Confidence interval.

## Data Availability

The SHARE data are provided free of charge to the scientific community and are available at http://share-project.org.
